# Epithelial Cell Dissemination and Readhesion: Analysis of Factors Contributing to Metastasis Formation in Breast Cancer

**DOI:** 10.5402/2012/601810

**Published:** 2012-03-12

**Authors:** Katya Hekimian, Sandra Meisezahl, Kristin Trompelt, Carola Rabenstein, Katharina Pachmann

**Affiliations:** ^1^Department of Experimental Heamatology and Oncology, Clinic for Internal Medicine II, Friedrich-Schiller-University Jena, 07747 Jena, Germany; ^2^Transfusion Center Bayreuth, D-95448 Bayreuth, Germany

## Abstract

Although considerable progress has been achieved in breast cancer diagnosis and treatment, the live-saving effect of mammography has hardly been measurable and the benefit of taxanes regarded as highly active is still a matter of debate, possibly because treatment effects have hitherto been mainly determined from the solid part of the tumor, due to lack of measurability of the systemic part of the disease. Here, we have quantified the influence on the systemic disease, cells mobilized from the solid tumor. Increased numbers of circulating epithelial cells were observed in screened individuals and still higher numbers in breast cancer patients with repeated mammograms as compared to mammogram naïve individuals. Taxanes as part of the subsequent systemic treatment led to mobilization of tumor suspect cells in up to 78% cases and the majority of relapses have occurred in these patients. Surgery-induced activation of disseminated cells may additionally contribute to metastasis formation.

## 1. Introduction

In most cancers it is not the primary tumor but the metastases which are responsible for fatal outcome. The prerequisites for metastasis formation and detection are as follows: (1) tumor cells must leave the tumor and get into the circulation; (2) these cells must evade destruction in the circulation and adhere in a distant organ; (3) the cells must grow to a metastasis of detectable size. Therefore it would be most promising to aim at detecting the tumor before it has been able to form metastases. Indeed, at least in breast cancer most tumors are detected when no measurable metastases are, yet, We added the highlighted part to the second address. present. However, a considerable proportion of patients during the further course of disease develop life-threatening metastases. Screening mammography, aiming at detecting early tumors has not yet shown convincing results [1, 2] and may even rather extend the period of disease than extending life time [3].

This may be due to the fact that cells can leave from the primary tumor when it is still below the limit of detection and it is not known at what time point these cells can settle and regrow. In addition, diagnostic and therapeutic approaches might contribute to mobilizing cells from the primary tumor since it has been shown that manipulations of the primary tumor may seed cells into the circulation [4–6].

In order to leave the tissue, cells need to detach from the surrounding cells which physiologically occurs during cell division. This may be one reason, why tumors with a high cell division rate have a higher metastatic capacity. During detachment cell adhesion molecules such as the Epithelial Cell Adhesion Molecule (EpCAM) are downregulated [7]. EpCAM is a cell surface adhesion protein that is frequently expressed at a high level on most solid tumor types, including prostate, breast, colon, gastric, ovarian, pancreatic, and lung cancer [8, 9]. It is detected at the basolateral membrane of the majority of epithelial tissues and is found to be overexpressed by a great variety of human adenocarcinomas and squamous cell carcinomas [10, 11]. Compared to primary and metastatic tissues the EpCAM expression has been reported to be approximately 10-fold lower on the cells shed into the circulation as part of the epithelial-mesenchymal transition, suggesting that loss of cell-cell adhesion is a prerequisite for tumor cell dissemination [12, 13]. However, silencing the EpCAM gene expression decreases the proliferation, migration, and invasion potential of breast cancer cell lines *in vitro* [11]. Cells may then be forced by intratumoral pressure into the lymphatics [14] and subsequently are drained into blood. Lymph nodes may be an effective barrier to cell clumps but less to individual cells [15]. In order to resettle and regrow which may occur in small vessels during slow down of blood flow and microthrombi formation [16], tumor cells seem to re-upregulate their EpCAM expression (mesenchymal-epithelial transition). However, tumor cells in blood may also mask surface structures preventing antibody binding [17–19] to evade the attack of the immune system and subsequent uncovering at the site of settling may contribute to readhesion of these cells.

We have, therefore, investigated the effect of diagnostic and therapeutic measures on the release of epithelial cells from normal and malignant tissue and the fate of the released cells in blood circulation.

## 2. Material and Methods

1 mL of anticoagulated peripheral blood was obtained, according to ethics committee approval and analyzed using the previously described microfluorimetric method, where assay method stability of the sample and reproducibility have been extensively described [20]. This volume was sufficient to detect cells in the presurgery as well as the postsurgery situation. In short, in order to compensate for shipping delays samples were subjected to red blood cell lysis at day 2 after blood drawing (usually with 95% viability) using 10 mL of erythrocyte lysis solution (Qiagen, Hilden, Germany) for 10 minutes in the cold, spun down at 700 g, and rediluted in 1 mL of PBS. 10 *μ*L of fluorescein-isothiocyanate-(FITC-) conjugated mouse anti-EpCAM (Milteny, Bergisch Gladbach Germany) and 1 *μ*L of phycoerythrin-(PE-) labelled anti-CD45 were added to 100 *μ*L of cell suspension, incubated for 15 minutes in the dark, readjusted to 1 mL and 20 *μ*L of this suspension were used for measuring epithelial-antigen-positive cells.

**Figure 1:**
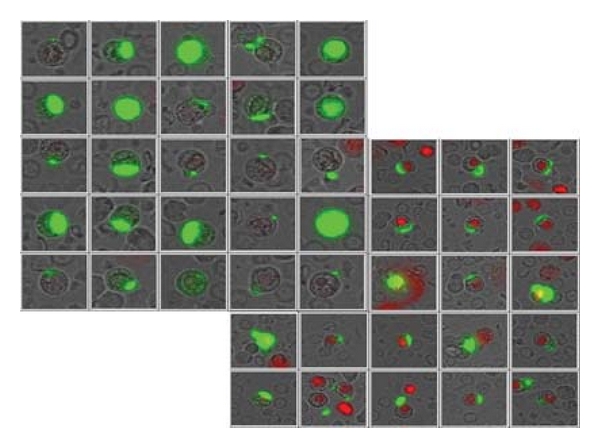
Gallery of vital (propidium iodide negative-red negative) and dead (propidium positive-red positive) EpCAM positive cells (green caps) provided by the automated microscopic system from a patient sample. Of note is the highly variable EpCAM expression on cells from an individual patient.

A defined volume of the cell suspension was applied to a defined area either on adhesion slides (Menzel Gläser, Braunschweig, Germany) or into wells of Elisa plates; the adherent cells were measured either using a Laser Scanning Cytometer (LSC Compucyte Corporation, Cambridge, MA, USA) and collecting the FITC-EpCAM and the PE-CD45 fluorescence using photomultipliers (PMT) or using image analysis in the ScanR (Olympus, Munich, Germany) which both gave equivalent results. Values are displayed in scatter grams and histograms. Both approaches enable the user to locate cells contained within the positive population for visual examination and to take photos and fluoromicrographs (Figure 1). Viability of the cells was visually detected and verified by Propidium Iodide (PI) staining (entering exclusively dying cells), looking for nuclear PI stain and surface EpCAM staining. Patients were analyzed for their circulating epithelial tumor cell (CETC) numbers before the actual mammography and followed during adjuvant treatment and neoadjuvant treatment. CETCs were analyzed at each visit if possible at intervals of three weeks. This allowed longitudinal followup of the CETCs. Cell numbers detected were up to thousandfold higher than the numbers detected by the CellSearch approach. The explanations for these discrepancies have been published [21]. These high cell numbers allowed monitoring of CETC during therapy. Patients were categorized according to the behavior of their CETC into those with tenfold decrease or increase. Statistical analyses were performed using the SPSS program, version 16.1.

## 3. Results

**Figure 2:**
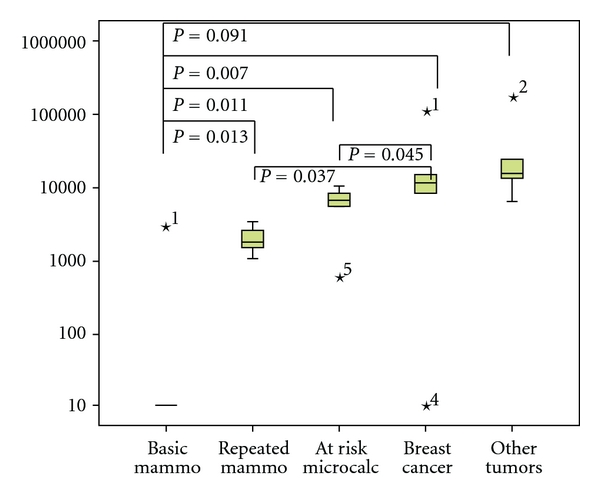
Box plots of the distribution of numbers of EpCAM-positive cells from a screening population of mammography naïve individuals before the basic mammogram, individuals with repeated mammograms without risk factors, individuals with repeated mammograms with risk factors (microcalcifications), from patients with confirmed breast cancer and from patients with other confirmed malignant tumors. Outliers are indicated by the statistical program as asterisks.

Mammography is the first diagnostic step taken in breast cancer screening and detection. Therefore the question whether this measure can contribute to mobilize epithelial cells was investigated. A screening population of 50 individuals without known mammary tumor and 20 patients with confirmed breast cancer were analysed for circulating epithelial cells before the actual mammogram. Only 5 individuals had had no prior mammography. 4 of them showed no circulating epithelial cells before the procedure and one individual had a moderate number of circulating epithelial cells (Figure 2). In contrast all 45 individuals who had had repeated mammograms showed circulating epithelial cells already before the procedure. Among these, 7 individuals with questionable findings (microcalcifications) had a 3-fold higher mean value and 5 individuals who had a history of prior malignancy of other organs than the breast had a tenfold higher mean value of circulating epithelial cells than the 33 individuals with no known malignancy. 20 patients with confirmed breast cancer had a 5-fold higher mean value (Figure 2). Circulating epithelial cells observed in these individuals may in part be due to previous mammographies having been squeezed out of the mammary gland by compression. Most surprisingly, obviously these cells were not eliminated immediately. The 20 patients with confirmed breast cancer in whom mammograms were performed at different instances during therapy had a 5-fold higher mean value than the supposedly healthy individuals with repeated mammograms (Figure 2).

After diagnosis the next step in breast cancer is the removal of the bulk of the tumor by surgery. We have already shown previously that surgery can lead to a surge in circulating epithelial cells, part of which may be tumor cells. After surgery the numbers of circulating epithelial cells can remain at an elevated level until systemic treatment which almost all patients receive before or after surgery, since it is assumed that breast cancer is a systemic disease already from very early on. In an attempt to further dissect the impact of such mobilized cells for final outcome the behaviour of the cells in the adjuvant therapy situation was compared to that in the neoadjuvant or primary chemotherapy situation, previously mainly reserved to patients with inoperable tumors but now increasingly applied also in operable tumors due to the possibility to perform breast conserving therapy. Neoadjuvant chemotherapy includes taxanes which have been shown to be highly effective against breast cancer cells and this was compared to the taxane-containing therapy given as adjuvant treatment in patients with increased risk (T2 tumors, involved lymph nodes or hormone receptor negative, HER2/neu ± tumors). In our institution taxane is given in three weekly cycles following 3 × FEC. In the group of patients treated with adjuvant chemotherapy circulating tumor cells were monitored in 70 patients. The tumor characteristics of the patients are given in Table 1.

**Figure 3:**
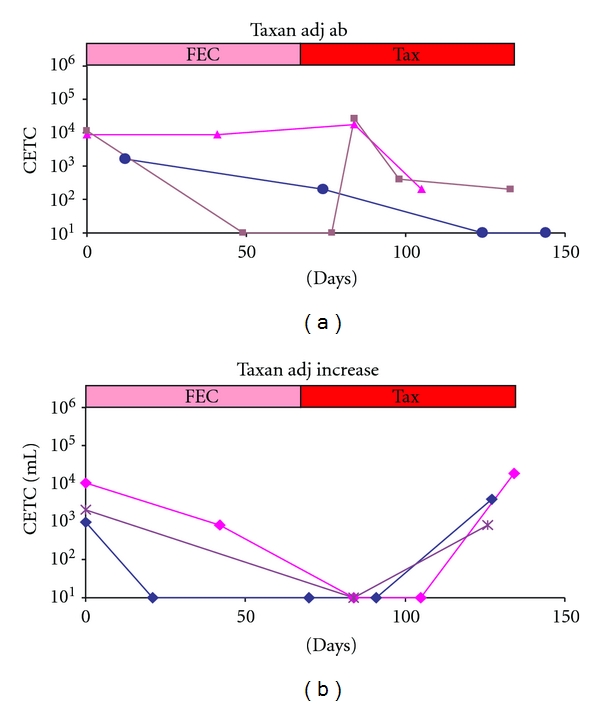
Changes in numbers of circulating epithelial tumor suspect cells (CETCs) during an adjuvant therapy schedule of FEC and taxane; (a) three typical examples of individual patients with a reduction in CETC and (b) three typical examples of individual patients with an increase during taxane treatment after previous decrease in CETC during FEC treatment.

Analyses were performed before treatment, before each new cycle and 3 weeks after the last cycle if possible (Figure 3(a), and 3(b)).

**Figure 4:**
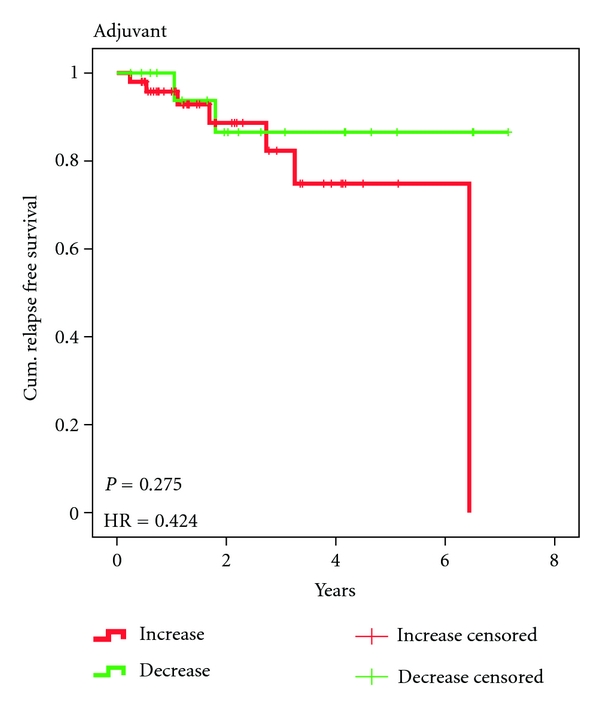
Kaplan-Meier relapse-free survival of 20 patients with decreasing CETC (green line) and 50 patients with increasing CETC (red line) during adjuvant therapy with FEC/Tax.

As has been reported before [22] the response to treatment in this patient group either was a decrease in cell numbers or an increase (almost always after an initial decrease) during treatment. Cell numbers decreased in 20 (29%) patients of which 2 patients (10%) suffered relapse and increased in 50 (71%) patients with 7(14%) relapses. In the 3 patients without involved lymph nodes (10%) relapses occurred only with increasing CETC, but relapses were more frequent (20%) in patients with involved lymph nodes and in patients with ER-negative tumors (23%) than with ER-positive tumors (11%). In the adjuvant situation in total 9/70 (13%) patients have relapsed until now. The Kaplan-Meier analysis is shown in Figure 4.

The Cox regression analysis revealed a nonsignificant hazard ratio of 0.42 in patients with decreasing CETC numbers as compared to patients with increasing CETC numbers. With a mean follow-up time of 2.2 years (0.25 to 6.52) the time in the adjuvant treatment group is too short to reveal a significant difference between patients with increasing and decreasing cell numbers.

CETC were monitored during neoadjuvant treatment in 60 patients before treatment, before each new cycle, and before surgery. The tumor characteristics of the 60 patients are given in Table 2.

**Figure 5:**
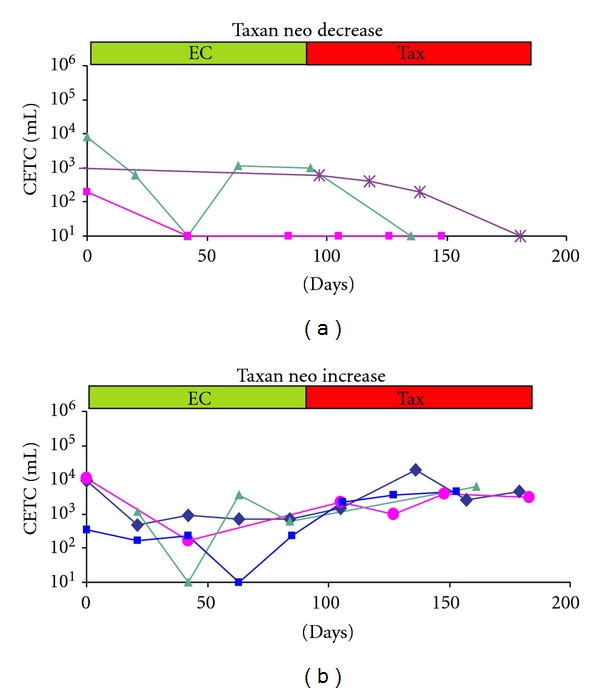
Changes in numbers of circulating epithelial tumor suspect cells (CETCs) during neoadjuvant therapy schedule of FEC and taxane; (a) three typical examples of individual patients with a reduction in CETC and (b) three typical examples of individual patients with an increase during taxane treatment after previous decrease in CETC during FEC treatment.

In the neoadjuvant treatment group, 13/60 (22%) patients showed a decrease (Figure 5(a)). Among the 13 patients with decreasing CETC none suffered relapse, whereas 16 (34%) of the 47 patients with increasing cell numbers (Figure 5(b)) have suffered relapse during the follow-up time with a median of 2.9 years (0.27 to 6.68).

**Figure 6:**
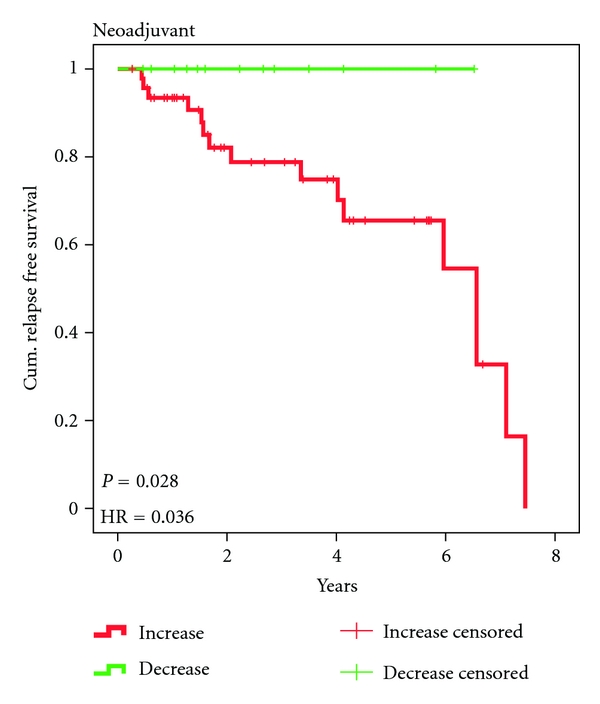
Kaplan-Meier relapse-free survival of 13 patients with decreasing CETC (green line) and 47 patients with increasing CETC (red line) during neoadjuvant therapy with FEC/Tax. Patients with increasing CETC versus the end of therapy have a 28-fold risk of relapse as compared with patients with decreasing CETC (*P* = 0.028).

The occurrence of relapses was more pronounced in patients with involved lymph nodes (66%) but more frequent in patients with ER-positive tumors (52%) and HER2/neu-negative tumors (53%) than in the adjuvant treatment group. In contrast to adjuvant treatment the difference in relapse-free survival in the Kaplan-Meier plot (Figure 6) between patients with decreasing and increasing CETC in the neoadjuvant treatment group was significant (*P* = 0.028) with a hazard ratio of 0.036 (95% confidence interval 0.022 to 1.63) and no plateau formation.

Thus in the neoadjuvant situation in total, 16/60 (27%) patients have relapsed and until now this has occurred exclusively in patients with circulating tumor cell numbers increasing in spite of chemotherapy. Tumor characteristics comprised more large tumors (72%) in the neoadjuvant treatment group versus the adjuvant treatment group (46%) but nodal involvement (50% versus 51%) and ER positivity (23% versus 22%) were comparable. HER2/neu-positivity was higher in the neoadjuvant treatment group (25% versus 11%). Total relapses in the patients treated with neoadjuvant therapy were twice as frequent as total relapses in the patients treated with adjuvant therapy (27% versus 13%).

## 4. Discussion

The fatal event in most solid cancers is metastasis formation for which a prerequisite is that cells leave the primary tumor and become adherent at distant sites before they grow and form metastases. These steps are still poorly understood. Although the optimal approach would be to detect the primary tumor before it starts seeding cells it is not sure whether this aim can ever be achieved. It is assumed that a tumor starts seeding cells from the 1 million cells level, a size of 1 mm when the tumor to date is neither unerringly detectable by mammography nor by other approaches.

Moreover, our investigations suggest that different diagnostic and therapeutic interventions can mobilize epithelial cells. Apart from naturally occurring dissemination of tumor cells from vascularization on and during tumor growth we here present indications that even the earliest diagnostic intervention, mammography, can mobilizes epithelial cells which, even under benign conditions, seem not to be rapidly cleared from the circulation. Numbers of circulating epithelial cells were below the threshold of detection only in mammography naïve women, whereas all individuals with repeated mammograms had significantly increased numbers of circulating epithelial cells, thus one can speculate that mammography leads to release of cells which can remain in the circulation for extended times comparably to the results reported on untransformed mammary cells in mice [23]. As long as these cells cannot perform the two subsequent steps of adherence and regrowth, this may remain without consequences. However, if an unknown malignant focus is present in the breast, mammography may contribute to early dissemination of malignant cells together with normal epithelial cells. Thus, we observed a threefold higher number of circulating epithelial cells in individuals with equivocal mammographic findings than in individuals without any signs of malignancy. Such mobilized cells with malignant traits may be able to settle and form metastases. This suggests the possibility that this may be one of the reasons for the mammographic paradox in young women with a higher frequency of aggressive breast cancers [1, 24] and contribute to the fact that the order of lives saved per individuals screened with mammography for breast cancer, is only around 1 per 1000—less than 0.1%, and that mammography screening by itself has little detectable impact on mortality due to breast cancer [2].

In patients monitored with mammography during the course disease of a known breast cancer numbers of circulating tumor suspect epithelial cells were significantly higher than in the two former populations which, in part may be due to surgery which also can lead to release of epithelial cells and may include normal as well as malignant cells [25]; therefore, in these patients the contribution of mammography is not clear.

Since almost all patients are treated subsequently with adjuvant chemotherapy and we have shown previously that also chemotherapy can lead to an increase in circulating epithelial tumor suspect cells [22, 26], it is difficult to discern the impact of the different manipulations. A comparison between adjuvant and neoadjuvant chemotherapy, in which surgery precedes or follows chemotherapy, may help to dissect the influence of the respective treatment. In order to have comparable treatment conditions we chose to investigate the influence of a taxane-containing chemotherapy before and after surgery. Increases were more frequent (50/60; 84%) during neoadjuvant therapy than during adjuvant treatment (50/70; 72%) and occurred frequently after an initial decrease during FEC treatment. The reincrease was observed almost invariably during taxan therapy. Although, also in the adjuvant treatment group the majority of relapses occurred in the patients with increasing cell numbers, this was exclusively the case in the neoadjuvant treatment group. How can these results be explained? Taxanes are highly effective drugs [27] but they also have dark sides: the good tumor reduction achieved in, for example, triple negative breast cancers [28, 29] may only in part be due to cell killing but at the same time to release of cells [26] due to intratumoral vessel decompression [14]. It has been reported that taxanes as microtubule-targeted drugs may enhance the ability of circulating tumor cells (CTCs) to endothelial engagement [30] during epithelial-to-mesenchymal transition and thus contribute to successful metastasis formation [31] of cells that are not eliminated. EpCAM is assumed to be downregulated during the journey of epithelial tumor cells in the circulation [32]. Our recent results, however, indicate that EpCAM rather may be masked probably by serum proteins [19]. Leukocytes and platelets, present at the site of microvascular cell arrest may contribute proteases which can lead to demasking of EpCAM and thus enhance adhesion to the endothelial wall.

The other question that arises is what the 2-fold higher frequency of relapses in the neoadjuvant treatment group as compared to the adjuvant treatment group is due to. One reason may be the larger size of the tumors; however, concentrations of disseminated cells did not surpass those observed during adjuvant treatment. Also, lymph node involvement was not different. HER2/neu-expression was more frequent in the neoadjuvant treatment group, but all HER2/neu-positive patients were additionally and/or subsequently treated with trastuzumab and relapses were nonsignificantly more frequent in patients with HER2/neu-negative than with HER2/neu-positive tumors. The main difference between the two treatment schedules is the timing of surgery. Surgery may help disseminated cells to escape [33] from their resting stage [34] due to the activity of different activating factors like Vascular Endothelial Growth Factor (VEGF) and cytokines, induced by the wound healing process.

It is noticeable that relapses in the neoadjuvant treatment group did not only occur early after surgery and showed no plateau but continued to occur even more than 5 years after the initial diagnosis. Careful review of the late relapses revealed that until now all the late relapses occurred in patients scheduled for tamoxifen therapy after surgery and who had finished their 5 years of hormone treatment. They had shown increasing numbers of tumor suspect cells after the end of hormone blocking therapy. Thus, cells which may already have settled and started to grow may have been stalled by hormone treatment and obviously were able to rapidly restart growing after the end of hormone blocking therapy. The increase in cell numbers in patients with confirmed breast cancer during different interventions and even after the end of therapy may, thus, be speculated to be a harbinger for recurrence of the disease.

## Figures and Tables

**Table 1:** Tumor characteristics of patients in complete remission and relapsed patients treated with adjuvant chemotherapy.

Adjuvant patients total 70							
	CR				Relapse		
		Decrease	Increase			Decrease	Increase
patients	61	18	43	Patients	9	2	7
*T* 1	31	11	20	*T* 1	5	0	5
*T* > 1	28	7	21	*T* > 1	4	2	2
*T* n.a.	2	0	2				

*N* 0	30	9	21	*N* 0	3	0	3
*N* > 0	30	9	21	*N* > 0	6	2	4
*N* n.a.	1	0	1	*N* n.a.			

*M* 0	61	18	43	*M* 0	9	2	7
ER pos	43	13	30	ER pos	5	2	3
ER neg	17	5	12	ER neg	4	0	4
ER n.a.	1	0	1	ER n.a.			

HER2/neu pos	15	5	10	HER2/neu pos	3	0	3
HER2/neu neg	44	12	32	HER2/neu neg	5	2	3
HER2/neu n.a	2	1	1	HER2/neu n.a.	1	0	1

(*T*: Tumor size, *N*: Lymph nodes, *M*: Metastases, ER: Estrogen receptor, n.a.: not analysed, CR: complete remission).

**Table 2:** Tumor characteristics of patients in complete remission and relapsed patients treated with neoadjuvant chemotherapy.

Neoadjuvant patients total 60							
	CR				Relapse		
		Decrease	Increase			Decrease	Increase

Patients	44	13	31	Patients	16	0	16
*T* 1	11	2	9	*T* 1	4	0	4
*T* > 1	31	9	22	*T* > 1	12	0	12
*T* n.a.	2	2	0	*T* n.a.			

*N* 0	24	7	17	*N* 0	4	0	4
*N* > 0	18	5	13	*N* > 0	12	0	12
*N* n.a.	2	1	1	*N* n.a.			

*M* 0	44	13	31	*M* 0	16	0	16
ER pos	21	3	18	ER pos	11	0	11
ER neg	21	9	12	ER neg	5	0	5
ER n.a.	2	1	1	ER n.a.			

HER2/neu pos	22	9	13	HER2/neu pos	6	0	6
HER2/neu neg	19	3	19	HER2/neu neg	10	0	10
HER2/neu n.a	3	1	2	HER2/neu n.a.	0	0	0

(*T*: Tumor size, *N*: Lymphnodes, *M*: Metastases, ER: Estrogen receptor, n.a.: not analysed, CR = complete remission).
